# Interaction between obesity and the *Hypoxia Inducible Factor 3 Alpha Subunit* rs3826795 polymorphism in relation with plasma alanine aminotransferase

**DOI:** 10.1186/s12881-017-0437-0

**Published:** 2017-07-28

**Authors:** Shuo Wang, Jieyun Song, Yide Yang, Yining Zhang, Nitesh V. Chawla, Jun Ma, Haijun Wang

**Affiliations:** 1 Institute of Child and Adolescent Health of Peking University, School of Public Health, Peking University Health Science Center, Beijing, 100191 China; 2 Interdisciplinary Center for Network Science and Applications (iCeNSA), University of Notre Dame, Notre Dame, IN 46556 USA; 3 Department of Computer Science and Engineering, University of Notre Dame, Notre Dame, IN 46556 USA; 4 Division of Maternal and Child Health, School of Public Health, Peking University Health Science Center, Beijing, 100191 China

**Keywords:** *Hypoxia inducible factor 3 alpha subunit*, Alanine aminotransferase, Single nucleotide polymorphisms, DNA Methylation, Obesity, Children

## Abstract

**Background:**

*Hypoxia Inducible Factor 3 Alpha Subunit (HIF3A)* DNA has been demonstrated to be associated with obesity in the methylation level, and it also has a Body Mass Index (BMI)-independent association with plasma alanine aminotransferase (ALT). However, the relation among obesity, plasma ALT, *HIF3A* polymorphism and methylation remains unclear. This study aims to identify the association between *HIF3A* polymorphism and plasma ALT, and further to determine whether the effect of *HIF3A* polymorphism on ALT could be modified by obesity or mediated by DNA methylation.

**Methods:**

The *HIF3A* rs3826795 polymorphism was genotyped in a case-control study including 2030 Chinese children aged 7–18 years (705 obese cases and 1325 non-obese controls). Furthermore, the *HIF3A* DNA methylation of the peripheral blood was measured in 110 severely obese children and 110 age- and gender- matched normal-weight controls.

**Results:**

There was no overall association between the *HIF3A* rs3826795 polymorphism and ALT. A significant interaction between obesity and rs3826795 in relation with ALT was found (*P*_inter_ = 0.042), with rs3826795 G-allele number elevating ALT significantly only in obese children (β’ = 0.075, *P* = 0.037), but not in non-obese children (β’ = −0.009, *P* = 0.741). Additionally, a mediation effect of *HIF3A* methylation was found in the association between the *HIF3A* rs3826795 polymorphism and ALT among obese children (β’ = 0.242, *P* = 0.014).

**Conclusion:**

This is the first study to report the interaction between obesity and *HIF3A* gene in relation with ALT, and also to reveal a mediation effect among the *HIF3A* polymorphism, methylation and ALT. This study provides new evidence to the function of *HIF3A* gene, which would be helpful for future risk assessment and personalized treatment of liver diseases.

**Electronic supplementary material:**

The online version of this article (doi:10.1186/s12881-017-0437-0) contains supplementary material, which is available to authorized users.

## Background

The prevalence of childhood obesity has witnessed a constant increase worldwide. In the year of 2013, 23·8% of boys and 22·6% of girls were overweight or obese in developed countries, and 12.9% of boys and 13.4% of girls were overweight or obese in developing countries [1].

Obesity could raise the risk of multiple co-morbid complications, including the Non-Alcoholic Fatty Liver Disease (NAFLD), type 2 diabetes, hypertension, cardiovascular disease, stroke and several kinds of cancers [2, 3]. It has been demonstrated by a meta-analysis study that the prevalence of childhood NAFLD is 7.6% in the general population, and could increase to 34.2% among obese children [4]. Usually used as a biomarker to reflect liver function clinically, the plasma alanine aminotransferase (ALT) is also reported to be associated with higher weight, body mass index (BMI) and waist circumference [5].

Evidence suggested that there could be a strong genetic background in the predisposition to both obesity [6] and NAFLD [7]. Meanwhile, epigenetics (heritable events not caused by changes in DNA sequence) could also contribute to the development of obesity [8] and NAFLD [9]. Being the most stable epigenetic marker, DNA methylation can not only be affected by the environment, but also modulated by genetic variants. It is reported that DNA methylation correlates with nearby Single Nucleotide Polymorphisms (SNPs), and studies conducted in various kinds of tissues (including brain, adipose, blood, et al.) have revealed that there are quantitative trait loci (QTLs) for DNA methylation, also called methylation QTLs (meQTLs) [10–12].

Previous studies have shed light on the association between *Hypoxia Inducible Factor 3 Alpha Subunit (HIF3A)* DNA methylation and obesity-related traits. Dick et al. [13] conducted an Epigenome-Wide Association Study (EWAS) among European white adults, uncovering a specific association between BMI and DNA methylation at 3 CpG (Cytosine-Phosphate-Guanine dinucleotides) sites of the *HIF3A* gene, and also reported nearby SNPs including the *HIF3A* rs3826795 polymorphism to be associated with methylation. In our previous study [14], we have reported that obese children had a relatively higher level of *HIF3A* DNA methylation, and *HIF3A* methylation had a BMI-independent association with plasma ALT. However, the relation among obesity, plasma ALT, *HIF3A* SNPs and methylation remains unclear.

In this paper, genotyping for the *HIF3A* rs3826795 polymorphism among 2030 Chinese children aged 7–18 years old was performed, and the *HIF3A* methylation data of 220 children detected in our previous study [14] was also used. The aims of this study are: (1) identifying the association between the *HIF3A* rs3826795 polymorphism and plasma ALT; (2) determining whether obesity could interact with rs3826795 on plasma ALT; and (3) investigating whether the effect of rs3826795 on ALT is mediated by *HIF3A* DNA methylation.

## Methods

### Subjects

We conducted a case-control study among 2030 Chinese children aged 7–18 years old from two independent study groups, including 705 obese cases and 1325 non-obese controls recruited from the urban regions of Beijing, China. The first study group came from the study on Adolescent Lipids, Insulin Resistance, and candidate genes (ALIR). The second study group was from the Comprehensive Prevention project for Overweight and Obese Adolescents (CPOOA). All obese individuals in the selected schools were recruited with their voluntary participation. The method of cluster sampling was adopted to recruit non-obese subjects from some classes of each grade in the same schools. The ALIR subjects were ascertained from adolescents aged 14–17 years in nine middle schools of Dongcheng District of Beijing, including 386 obese adolescents and 551 non-obese adolescents. The CPOOA subjects were recruited from children and adolescents aged 7–18 years old in five elementary and middle schools of the Haidian District of Beijing, comprising 319 obese children and adolescents and 774 non-obese children and adolescents. The ascertainment strategies for the two study groups have been described in detail previously [15, 16]. We used the uniform BMI percentile criteria for obese and non-obese children, which were determined in a representative Chinese population [17]. According to the criteria, the children and adolescents with an age- and gender-specific BMI ≥ 95th percentile are defined as obese, whereas those with a BMI between 15th and 95th percentile are non-obese. The individuals with any cardiovascular or metabolic disease were excluded. Anthropometric measurements, including height and weight, were measured at school according to standard protocols. Fasting venous blood samples were taken for detection of ALT.

Methylation data were collected from 110 severely obese children and 110 normal-weight age- and gender-matched controls, which were chosen from the CPOOA study. We chose those with an age- and gender-specific BMI ≥ 97th percentile as the severely obese cases, and those with BMI between 15th and 85th percentile as non-obese controls. We point the reader to our prior work, where we have described the study design for the methylation detection [14].

Studies were approved by the Ethic committee of Peking University Health Science Center. Written informed consent was provided by all participants and, in the case of minors, by their parents.

### SNP genotyping

The *HIF3A* rs3826795 polymorphism was genotyped using genomic DNAs extracted from blood leukocytes by the phenol-chloroform extraction method. Genotyping was conducted on MassARRAY System (Sequenom, San Diego, CA, USA). Primers, including a pair of amplification primers and an extension primer, were designed with Sequenom MassArray Assay Design Suite. A multiplex polymerase chain reaction was performed, and unincorporated double stranded nucleotide triphosphate bases were dephosphorylated with shrimp alkaline phosphatase followed by primer extension. The purified primer extension reaction was spotted on to a 384-element silicon chip (SpectroCHIP, Sequenom) and analyzed in the Matrix assisted laser desorption ionization time of flight mass Spectrometry (MALDI-TOF MS, Sequenom). The resulting spectra were processed with MassArray Typer (Sequenom) (http://www.sequenom.com). The genotyping call rate of the *HIF3A* rs3826795 polymorphism was 97.9%. All the experiments were done by investigators who were blind to the phenotypes.

### DNA methylation detection

Details of the methylation examination were described previously [14]. The *HIF3A* DNA methylation data were collected from genomic DNA of peripheral blood leukocytes among 110 obese children and 110 non-obese controls. The blood samples were the same for SNP genotyping and methylation detection. We used Sequenom’s MassARRAY system (Sequenom, San Diego, CA) to perform quantitative methylation analyses [18]. A fully-methylated positive control and a 0-methylated negative control were included for each run. Methylation detection was conducted in duplicate for all the samples. Three samples failed to give a reliable PCR product, and 5 samples were excluded from analysis samples because of low call rates (<80%). Thus, we obtained data of 9 CpG sites (located form 46,801,557 to 46,801,760) of *HIF3A* DNA methylation among 107 obese children and 105 controls.

### Statistical analyses

Quanto software (University of Southern California, Los Angeles, CA) was used to conduct power analysis. As no study has previously reported the interaction between obesity and the *HIF3A* rs3826795 polymorphism on ALT, we could only estimate the sample size. Using the additive genetic model, at a two-sided significance level of *P* < 0.05, with effect allele frequency of 0.41 (referring to the risk allele frequency of Singapore population [19]), with the proportion of obese children as 0.35 [20], assuming the effects on logALT of *gene*, *obesity* and *gene × obesity* were 0.10, 0.30 and 0.20 respectively, 1906 people were estimated to obtain a statistical power of 85%.

The genotype data were tested for deviation from Hardy-Weinberg equilibrium. The polymorphism was analyzed under additive models. For the values of ALT, we used the log converted values (logALT) because of the skewed distribution of the original values, and then we used the criterion of Mean ± 3SD and excluded 26 extreme values. Differences in general characteristics between obese and non-obese subjects were tested with Chi-square (categorical variables) or t-test (continuous variables). Linear regression models adjusting for *age*, *age* ^2^ and *gender* were used to examine the effect of SNP on logALT. In order to fully control the effect of age on ALT, age and age^2^ were adjusted for simultaneously, since phenotypes could be highly influenced by age especially in the children population, and the association between the phenotype and age could be non-linear. The method of age-squared adjustment was also used in previous studies [21, 22]. Interaction analyses were conducted using stratified analyses and the statistical tests for the interaction term.

The mediation analysis was based on the model brought forward by Baron, et al. [23], and was used by other literatures, especially in the domain of psychology [24]. Three multivariate linear regression models were conducted, all adjusting for age, age^2^ and gender: (1) *Y = cX + p*_*1*_ *age + q*_*1*_ *age* ^2^ *+ r*_*1*_ *gender + e*_*1*_; (2) *M = ax + p*_*2*_ *age + q*_*2*_ *age* ^2^ *+ r*_*2*_ *gender + e*_*2*_; (3) *Y = c*_*r*_ *X + bM + p*_*3*_ *age + q*_*3*_ *age* ^2^ *+ r*_*3*_ *gender + e*_*3*_. The statistical test of the mediation effect included several steps: (1) the association between the independent variable and the dependent variable was tested (the coefficient *c*); (2) the association between the independent variable and the potential mediator was tested (the coefficient *a*); (3) both of the independent variable and the potential mediator were entered simultaneously as predictors of the dependent variable, and the coefficient *b* was tested to establish the significance of the mediation effect; (4) if the mediation effect was significant, the type of mediation effect could be determined by testing the coefficient *c*_*r*_, indicating either full mediation effect (*c*_*r*_ not significant) or partial mediation effect (*c*_*r*_ still significant).

A two-sided *P* < 0.05 was considered as nominally significant. Statistical analyses were performed using SPSS 18.0 software (SPSS Inc., Chicago, IL).

## Results

### General characteristics of the study groups

General characteristics of obese cases and non-obese controls are summarized in Table 1 (Dataset in Additional file 1). The age of the participants ranged from 7 to 18 years old, with the average age of 12.9 ± 2.7 years old. We found no significant difference in age between obese and non-obese groups (*P* > 0.05), but there was a higher proportion of boys in the obese group than in the non-obese group (*P* < 0.001). Compared to non-obese children, obese children had higher plasma ALT levels (*P* < 0.001). The difference of ALT between obese and non-obese groups remained significant after adjusting for *age*, *age* ^2^ and *gender* (*P* < 0.001). Details of the characteristics of the 110 extremely obese children and 110 matched controls with methylation examination were described previously [14]. Briefly, there was no difference between the two groups in age (*P* = 0.934) or gender (*P* = 0.946), and the 110 extremely obese children had a higher level of ALT as compared with controls (*P* < 0.001).

**Table 1:** General characteristics of the study groups

	Total	Obese	Non-obese	*P*
Number	2030	705	1325	
Boys (%)	1218(60.0)	484(68.7)	734(55.4)	<0.001
Age (year)	12.9 ± 2.7	12.8 ± 2.6	12.9 ± 2.7	0.497
Body mass index (BMI, kg/m^2^)	23.8 ± 4.8	28.1 ± 3.9	21.5 ± 3.5	<0.001
Alanine aminotransferase (ALT, IU/L)	1.12 ± 0.27	1.26 ± 0.29	1.05 ± 0.24	<0.001

Values are provided as Mean ± SD if not indicated otherwise

For ALT, the log converted values are used instead of the original values

### Interaction analyses of obesity and the *HIF3A* rs3826795 polymorphism on plasma ALT levels

The risk allele (G allele) frequency of the *HIF3A* rs3826795 polymorphism was 48.3% in the current study. The genotype distribution of polymorphism in the control group was in Hardy-Weinberg equilibrium (*P* > 0.05). We first examined the association between the variant and obesity, but found that rs3826795 was not significantly associated with obesity (*P* = 0.843), or BMI (*P* = 0.268) (both adjusting for age, age^2^ and gender). We then divided the study population by rs3826795 genotype and obesity group (obese vs non-obese), and compared the ALT levels in different subgroups (Table 2). The association between ALT and the *HIF3A* rs3826795 genotype was not significant in the overall population (*P* = 0.340). By conducting stratified analyses, we found that the number of *HIF3A* rs3826795 G-allele was positively associated with elevated plasma ALT in obese children (β’ = 0.075, *P* = 0.037), with each G-allele increasing the logALT level by 0.030 (which was the unstandardized coefficient of SNP in the regression model). We observed no significant association between ALT and the *HIF3A* rs3826795 genotype in non-obese children (*P* = 0.741). Using a multivariate linear regression model, we then tested the interaction term (*SNP × obesity group*), and found a significant interaction between G-allele number in the *HIF3A* rs3826795 polymorphism and obesity on the plasma ALT (*P* = 0.042).

**Table 2:** Interaction analyses of the *HIF3A* rs3826795 polymorphism and obesity on plasma ALT

	Mean ± SD of logALT (IU/L)					
	AA (*n* = 535)	AG (*n* = 985)	GG (*n* = 468)	β’	*P*	*P*_*inter*_
Total	1.11 ± 0.27	1.12 ± 0.27	1.14 ± 0.28	0.020	0.340	
Non-obese	1.04 ± 0.25	1.06 ± 0.23	1.05 ± 0.23	−0.009	0.741	0.042
Obese	1.24 ± 0.26	1.25 ± 0.30	1.30 ± 0.29	0.075	0.037	

β’ stands for the standardized coefficient of G allele number in the model

*P*_inter_ stands for the *P* value of the interaction term in the linear regression model, including logALT as the dependent variable, and SNP, obesity group, SNP × obesity group, age, age^2^ and gender as independent variables

*HIF3A*: *Hypoxia Inducible Factor 3 Alpha Subunit*; *ALT* alanine aminotransferase

### Mediation analysis of the *HIF3A* rs3826795 polymorphism, methylation and plasma ALT

*HIF3A* DNA methylation was tested in 110 children with severe obesity and 110 non-obese age- and gender- matched controls. We used the data of CpG unit at 46801699 to conduct the mediation analysis among *HIF3A* SNP, methylation and ALT.

**Fig. 1:**
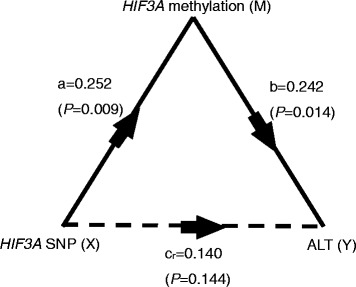
Mediation analysis in obese children for *HIF3A* SNP, methylation and plasma ALT. ALT: alanine aminotransferase; *HIF3A*: Hypoxia Inducible Factor 3 Alpha Subunit; SNP: Single Nucleotide Polymorphism. In order to distinguish among the three linear regression models constructed in the mediation analysis, the coefficients in the model were represented b*y ‘a’,’ b’, ‘c’ and ‘c*_*r*_ *’. T*he first regression model used *‘c’* as the coefficient of SNP in association with ALT; the second model used ‘*a’* as the coefficient of SNP in association with methylation; the third model put methylation and SNP as independent variables simultaneously, and used *‘b’* and *‘c*_*r*_ *’* as the coefficients in association with ALT. *‘X’*, *‘Y’* and *‘M’* were used to represent the independent variable, the dependent variable and the mediator in the mediation analysis model

We used a mediation model to examine whether the *HIF3A* methylation acted as a mediator in the association between the *HIF3A* rs3826795 polymorphism and plasma ALT, as shown in Fig. 1. Here *‘a’*, *‘b’*, *‘c’* and *‘c*_*r*_ *’* were used to represent the coefficients in the mediation analysis model. In the 110 obese children with methylation data, the *HIF3A* rs3826795 G-allele number was positively associated with elevated ALT (*c* = 0.203, *P* = 0.034), with each G-allele increasing the logALT level by 0.085. There was a positive association between the *HIF3A* rs3826795 G-allele number and *HIF3A* methylation (*a* = 0.252, *P* = 0.009), with each G-allele increasing the methylation level by 0.032. By testing ‘b’, we found the mediation effect was significant (*b* = 0.242, *P* = 0.014), and the insignificance of ‘*c*_*r*_’ indicated a complete mediation effect (*c’* = 0.140, *P* = 0.144), meaning that the effect between SNP and ALT in obese children would not be significant without methylation as the mediator. Further calculation (*ab/c* × 100%) reflected the mediation effect was 70.0%, meaning that 70.0% of the total effect between SNP and ALT was mediated by DNA methylation.

Further, we conducted analyses in the 110 non-obese children, and found no significant association between any two variables among the *HIF3A* rs3826795 polymorphism, *HIF3A* methylation and ALT (*P* > 0.05). No association was observed between rs3826795 and the methylation of the CpG site at 46801699 (β’ = 0.198, *P* = 0.070), while there was no significant association between the *HIF3A* rs3826795 and ALT (β’ = 0.023, *P* = 0.829), or between *HIF3A* methylation and ALT (β’ = 0.146, *P* = 0.160).

## Discussion

To the best of our knowledge, our study is the first to find that 1) the *HIF3A* rs3826795 polymorphism interacts with obesity on plasma ALT, with the rs3826795 G-allele number elevating the ALT level only in obese children; and 2) the effect of the *HIF3A* rs3826795 polymorphism on ALT in obese children is mediated by *HIF3A* DNA methylation.

*HIF3A* encodes for the protein of HIF-3α, which is the α-3 subunit of the hypoxia inducible transcription factors (HIFs). Uncovered in 1992, HIF acts as a heterodimeric transcription factor composing a β subunit (HIF-β) and one of three α subunits (HIF-1α, HIF-2α, HIF-3α), and regulates many adaptive responses to low oxygen tension (hypoxia) on both cellular and physiological levels [25]. HIF-3α has not been investigated as thoroughly as the other α subunits, but it is usually regarded as a negative regulator of HIF-1α and HIF-2α [26]. The *HIF3A* gene locates at 19q13.2 with a length of 43 kb, containing 19 introns and 8 kinds of alternative splicing [27].

The risk allele frequency of the *HIF3A* rs3826795 polymorphism was 0.79–0.82 in white adults of the EWAS study, and another study [28] focusing on *HIF3A* variant and weight change reported the risk allele frequency of rs3826795 to be 0.82–0.83 in US adults of European ancestry. Pan et al. [19] examined in Singapore mother–offspring pairs and reported the risk allele frequency of rs3826795 to be 0.41. In the current study we found that the risk allele frequency of rs3826795 was 0.48 in Chinese children, which is lower than that in the population of European ancestry, and similar to Singapore population.

The rs3826795 polymorphism and CpG site 46,801,699 both locate at the first intron of the *HIF3A* gene, with the CpG site about 1.3 kb downstream rs3826795. In the current study we found that the *HIF3A* rs3826795 polymorphism could affect the *HIF3A* methylation at CpG site 46,801,699 and other nearby CpG sites, indicating rs3826795 as a methylation quantitative-trait locus (metQTL). Known to be influenced by environmental factors, DNA methylation can also be modulated by genetic variants. Grundberg et al. [10] conducted analysis of metQTL in a genome study and revealed that 28% genome CpGs were associated with nearby SNPs (within 100 kb). The study further identified that meQTLs over-lapping metabolic disease loci were enriched in genetic enhancer. Another study conducted by Voisin et al. [12] also found that meQTLs tended to locate in intergenic regions, showing that obesity-associated SNPs were underrepresented in promoters but enriched in intergenic regions.

In the EWAS study, Dick et al. [13] reported the *HIF3A* rs3826795 polymorphism to be associated with methylation at a nearby CpG site (46801642), with each risk allele (G allele) increasing the methylation level by 0.039–0.051 in different cohorts. The positive association between rs3826795 and methylation at CpG site 46,801,642 was also found in the current study, with each risk allele increasing its methylation by 0.029 in obese children, and 0.022 in non-obese children.

Several previous studies showed that *HIF3A* methylation was associated with BMI. Through the EWAS study of methylation patterns in peripheral blood DNA in one discovery cohort and two replication cohorts, Dick et al. [13] uncovered a specific association between increased BMI and higher methylation levels of 3 CpG sites at the first intron of *HIF3A*. Demerath et al. [29] validated the results through another EWAS study, and found that *HIF3A* methylation was associated with 30-year period BMI change in African American adults. Pan et al. [19] found that higher methylation levels at *HIF3A* CpGs were associated with greater infant weight and adiposity, and further tested an interaction between birth weight and rs3826795 with *HIF3A* methylation as outcome. Neither study [13, 19] found significant associations between *HIF3A* SNP and BMI or birth weight, which is in consistency with the current study. In the current study, by testing the nearby SNP and conducting interaction analysis and mediation analysis, we identified that the *HIF3A* rs3826795 polymorphism interacts with obesity on ALT, and *HIF3A* DNA methylation could act as a mediator in the effect of rs3826795 on ALT in obese children. This finding provided evidence on the function of *HIF3A* gene.

The mechanism by which the *HIF3A* gene could lead to elevation of plasma ALT in obese children is unknown. ALT is clinically used to reflect the defect of liver function, which is related to hepatotoxic fatty acids increase caused by the visceral adipose deposition [30]. ALT elevation is shown to be strongly associated with central adiposity and related features including dyslipidemia, diabetes and hypertension [31]. In a review focusing on obesity and inflammation, Johnson et al. [32] described that in the status of obesity, a variety of cell populations began to exhibit an increased adipose mass and adipocyte diameter, which could lead to cellular hypoxia and further result in pro-inflammatory cytokine production. Rausch et al. [33] found that obesity in male C57BL/6 J mice was associated with increased expression of HIF, and suggested that hypoxia could be a potential contributor to the local and generalized inflammatory state. Chronic low-grade inflammation from visceral adipose tissue is considered as one of the two of the most critical factors (another factor is excess free fatty acids) contributing to liver injury progression. Thus, a possible speculation could be that obesity started a cellular hypoxia status through expanded adipocyte size, and inflammatory cytokines are increased by hypoxia, resulting in the defect of liver function and elevated ALT.

Several limitations of the current study should be noticed. First of all, though we have tested the relation among obesity, ALT, *HIF3A* SNP and methylation by conducting the interaction analysis and the mediation analysis, the case-control design of the current study means that we cannot assess the causality. However, the identified association provided evidence for further studies. Secondly, known to be a tissue specific biomarker, the *HIF3A* methylation levels were examined in the current study using peripheral blood samples instead of adipose issues or hepatic issues. As reported by Pfeiffer et al., *HIF3A* mRNA expression was higher in subcutaneous adipose tissue as compared with visceral adipose tissue, and correlated with parameters of adipose tissue dysfunction [34]. However, several other studies also used peripheral blood to test DNA methylation [12, 29], and supported the use of whole-blood DNA methylation profiling for identification of relevant epigenetic changes. Thirdly, gene-environment interaction analysis and mediation analysis usually need a large sample size. Although only one polymorphism was tested in the current study, multiple tests in the interaction analysis and the mediation analysis have been performed, therefore the result should be interpreted with caution. Besides, for the SNP data, there was a higher proportion of boys in the obese group than in the non-obese group. Although we adjusted for gender in the regression model, the different proportion of male subjects may still influence the result of association. Finally, we did not test cytokines or gene expression data other than DNA methylation. Further studies were needed for understanding the mechanism of the relation among obesity, ALT and the *HIF3A* gene, and also the mechanism of *HIF3A* meQTL.

## Conclusion

In conclusion, we found that obesity interacts with the *HIF3A* rs3826795 polymorphism on plasma ALT, and the effect of rs3826795 on ALT in obese children could be mediated by *HIF3A* DNA methylation. The results provided new evidence to the function of the *HIF3A* gene and the mechanism linking obesity and ALT. The study would be helpful for future risk assessment and personalized medicine of liver diseases such as NAFLD.

## Additional file

Additional file 1: Dataset of *HIF3A* polymorphism, methylation, obesity and ALT. In this dataset, each row represents a case and each column represents a variable. This dataset includes 6 variables: (1) log converted plasma ALT; (2) rs3826795 genotype (‘0’ = ‘AA’, ‘1’ = ‘AG’, ‘2’ = ‘GG’); (3) obesity status (‘1’ = ‘non-obese’, ‘2’ = ‘obese’); (4) age_range (years); (5) DNA methylation at 46801699. (XLS 49 kb)

## Abbreviations

ALT: Alanine aminotransferase
CpG: cytosine-phosphate-guanine dinucleotides
EWAS: Epigenome-Wide Association Study
*HIF3A*: *Hypoxia Inducible Factor 3 Alpha Subunit*
meQTLs: methylation QTLs
NAFLD: Non-alcoholic fatty liver disease
QTLs: Quantitative trait loci
SNP: Single nucleotide polymorphism
